# Supplementary material for: Degradation and Lifetime Prediction of Epoxy Composite Insulation Materials under High Relative Humidity
Source: Polymers (Basel). 2023 Jun 13;15(12):2666. doi: 10.3390/polym15122666 (PMC10304519; doi:10.3390/polym15122666)
Supplement: Supplementary file 1 [file polymers-15-02666-s001.zip › polymers-2448228-SM.pdf]

Supplementary Materials

Degradation and Lifetime Prediction of Epoxy Composite Insulation Materials under High Relative Humidity

Table S1. Material properties versus aging hours under different aging conditions.

**95 °C & 95% RH**	**0h**	**180h**	**420h**	**660h**	**1000h**		
**Modulus (GPa)**	**2.288 ± 0.115**	1.970 ± 0.025	2.495 ± 0.272	2.263 ± 0.193	1.967 ± 0.350		
Mechanical strength (MPa)	69.1 ± 6.4	40.0 ± 14.2	49.7 ± 10.2	36.2 ± 5.3	34.2 ± 6.3		
Strain at break (mm/mm)	0.072 ± 0.008	0.025 ± 0.006	0.030 ± 0.005	0.020 ± 0.003	0.023 ± 0.006		
Thermal conductivity (W/m/K) at 25 °C	0.75 ± 0.02	0.66 ± 0.02	0.82 ± 0.06	0.72 ± 0.06	0.73 ± 0.01		
Heat capacity (J/kg/K) at 25 °C	1027 ± 27	871 ± 20	1031 ± 5	972 ± 9	933 ± 11		
Coefficient of linear thermal expansion (µm/m/°C) 40 - 90 °C	33.7 ± 1.6	21.5 ± 6.3	16.9 ± 4.6	23.9 ± 1.4	19.3 ± 5.4		
Glass transition temperature (T_g, °C)	116 ± 6	104 ± 4	102 ± 1	103 ± 3	102 ± 1		
Mass change (%)	0	0.64 ± 0.08	0.74 ± 0.07	0.67 ± 0.12	0.77 ± 0.10		
Density (kg/m^3) at 25 °C	1811 ± 8	1877 ± 38	1721 ± 63	1866 ± 33	1842 ± 5		
Relative ester C=O absorption	4.775 ± 0.092	2.150 ± 0.263	1.781 ± 0.077	1.467 ± 0.168	1.078 ± 0.030		
Mass loss at 900 °C under nitrogen (%)	40.71	39.01	36.92	38.06	40.04		
Decomposition temperature at 10 K/min (T_d, °C)	412	410	406	407	411		
**85 °C & 95% RH**	**0h**	**185h**	**501h**	**720h**	**1010h**	**1509h**	**2012h**
Modulus (GPa)	2.288 ± 0.115	1.962 ± 0.235	2.716 ± 0.250	2.606 ± 0.166	2.494 ± 0.059	2.164 ± 0.107	2.444 ± 0.505
Mechanical strength (MPa)	69.1 ± 6.4	59.8 ± 5.3	60.0 ± 3.4	50.1 ± 3.2	50.3 ± 4.7	43.0 ± 3.0	34.5 ± 5.0
Strain at break (mm/mm)	0.072 ± 0.008	0.067 ± 0.011	0.041 ± 0.007	0.035 ± 0.004	0.024 ± 0.003	0.029 ± 0.008	0.034 ± 0.021
Thermal conductivity (W/m/K) at 25 °C	0.75 ± 0.02	0.85 ± 0.11	0.81 ± 0.10	0.73 ± 0.10	0.71 ± 0.02	0.59 ± 0.06	0.67 ± 0.06
Heat capacity (J/kg/K) at 25 °C	1027 ± 27	1129 ± 98	1020 ± 101	1078 ± 168	977 ± 26	855 ± 25	918 ± 58
Coefficient of linear thermal expansion (µm/m/°C) 40 - 90 °C	33.7 ± 1.6	34.7 ± 2.9	30.7 ± 0.3	25.0 ± 4.6	24.8 ± 4.6	29.4 ± 3.0	46.7 ± 7.3
Glass transition temperature (T_g, °C)	116 ± 6	112 ± 2	111 ± 2	110 ± 2	108 ± 1	105 ± 3	105 ± 4
Mass change (%)	0	0.53 ± 0.05	0.64 ± 0.10	0.60 ± 0.05	0.82 ± 0.33	0.86 ± 0.12	0.86 ± 0.04
Density (kg/m^3) at 25 °C	1811 ± 8	1807 ± 8	1830 ± 13	1768 ± 14	1786 ± 11	1764 ± 17	1732 ± 26
Relative ester C=O absorption	4.775 ± 0.092	2.943 ± 0.045	2.240 ± 0.243	1.999 ± 0.233	1.733 ± 0.253	1.448 ± 0.259	1.127 ± 0.343
Mass loss at 900 °C under nitrogen (%)	40.71	39	38.49	37.62	33.03	39.72	37.88
Decomposition temperature (T_d, °C)	412	403	395	393	399	417	418
**75 °C & 95% RH**	**0h**	**502h**	**1006h**	**1511h**	**2013h**		
Modulus (GPa)	2.288 ± 0.115	2.012 ± 0.496	2.541 ± 0.148	1.184 ± 0.486	2.451 ± 0.299		
Mechanical strength (MPa)	69.1 ± 6.4	56.3 ± 6.5	58.3 ± 9.1	53.8 ± 4.5	49.1 ± 7.5		
Strain at break (mm/mm)	0.072 ± 0.008	0.057 ± 0.019	0.028 ± 0.003	0.083 ± 0.009	0.035 ± 0.024		
Thermal conductivity (W/m/K) at 25 °C	0.75 ± 0.02	0.69 ± 0.03	0.70 ± 0.05	0.62 ± 0.05	0.70 ± 0.04		
Heat capacity (J/kg/K) at 25 °C	1027 ± 27	958 ± 63	952 ± 17	826 ± 19	965 ± 74		
Coefficient of linear thermal expansion (µm/m/°C) 40 - 90 °C	33.7 ± 1.6	31.0 ± 12.6	31.6 ± 5.0	61.2 ± 11.2	42.9 ± 7.8		
Glass transition temperature (T_g, °C)	116 ± 6	110 ± 2	108 ± 1	99 ± 13	100 ± 10		
Mass change (%)	0	0.67 ± 0.09	0.68 ± 0.12	0.66 ± 0.19	0.78 ± 0.20		
Density (kg/m^3) at 25 °C	1811 ± 8	1854 ± 72	1767 ± 60	1816 ± 14	1743 ± 52		
Relative ester C=O absorption	4.775 ± 0.092	3.191 ± 0.348	3.688 ± 0.434	2.953 ± 0.441	2.743 ± 0.614		
Mass loss at 900 °C under nitrogen (%)	40.71	39.26	38.49	38.96	36.63		
Decomposition temperature (T_d, °C)	412	415.5	415.5	418.7	417.9		
